# Can rising labor costs boost private sector R&D investment? : Evidence from a survey of Chinese private firms

**DOI:** 10.1371/journal.pone.0268287

**Published:** 2022-08-04

**Authors:** Chuyao Deng, Yang Liu, Doudou Gu

**Affiliations:** 1 School of Economics, Harbin University of Commerce, Harbin, Heilongjiang, China; 2 School of Economics, Wuhan University, Wuhan, China; Sichuan Agricultural University, CHINA

## Abstract

This paper constructs a double difference model (DID) based on the China Private Enterprise Survey (CPES) data over the period 1995–2019, combined with the 2005 national census data and considering the policy shock of the implementation of the Chinese government’s Minimum Wage Regulation in March 2004, to investigate whether rising labor costs promote private firms’ innovation investment. Robustness tests are conducted using placebo tests and event study methods. The study finds that (1) rising labor costs significantly increase private firms’ R&D investment and that this effect has significant lag and cumulative effects; (2) private industrial firms (especially above-scale private industrial firms) are more affected by rising labor costs than other private firms and have more incentives to increase innovation investment; and (3) innovation investment of below-scale private industrial firms is not significantly affected by rising labor costs.

## I. Introduction

This paper explores whether firms increase input factors, such as capital for innovation and competitiveness, when labor costs rise. On the one hand, several scholars have argued that increasing labor costs promote firm innovation [1–3]. First, according to neoclassical economics, a relative increase in the price of labor factors will prompt firms to increase other input factors, such as capital, given that labor and capital are the two primary factors of production [4, 5]. When there is no smooth substitution between factors, firms are incentivized to create or adopt new production conditions and increase labor productivity to reduce unit labor costs [6, 7]. Second, rising labor costs may promote technological innovation through human capital accumulation in the face of increasing labor cost forces. However, firms may hire fewer new employees as they increase the training of existing employees and improve the quality of their human capital [8, 9].

On the other hand, higher labor costs may be detrimental to firm innovation [10–12]. The increase in labor costs strains the financial position of firms, especially labor-intensive firms, and the rise in labor expenditures may force them to miss out on many NPV-positive investments to fill the capital gap caused by the increase in labor costs, i.e., the rise in labor costs has a "crowding-out effect" on firms’ innovation inputs [13]. Given the different theoretical expectations, whether rising labor costs promote or hinder firm innovation needs to be addressed through empirical testing.

In terms of empirical evidence, most studies have concluded that rising labor costs promote firm technological innovation, but there is heterogeneity across industries, regions, and firms [10, 14, 15]. Most Chinese studies use data from the Chinese industrial enterprise database and listed companies. Their research subjects are primarily state-owned and above-scale nonstate-owned large and medium-sized industrial enterprises. At the same time, less attention is given to private enterprises and below-scale enterprises. Thus, they cannot comprehensively discuss the impact of rising labor costs on firm innovation. In addition, in terms of research methodology, only a few Chinese scholars have conducted studies from the perspective of minimum wage, and the results vary [16–18]. Therefore, additional research on this topic still needs to be conducted.

This paper attempts to make a marginal contribution to the existing literature in the following ways. For example, there is still room for further expansion in empirical research to explore the effect of rising labor costs using the minimum wage on firm innovation and its mechanisms. First, given the existence of numerous theories and different expectations resulting from the means of influence in academia, we need to empirically examine whether rising labor costs promote or hinder firm innovation, plus the fact that only a few scholars have studied the impact of rising labor costs on firm innovation from the perspective of the minimum wage. There is an urgent need for additional research with different findings on this topic. Second, the current research on labor costs in China has been very positive. Most studies on rising labor costs in enterprise innovation in China have used data from the Chinese Industrial Enterprise Database and listed companies. They have focused on state-owned and nonstate-owned large and medium-sized industrial enterprises above a specific size but less on private enterprises and enterprises below a particular size. As there are many small enterprises in China, measuring the impact of rising labour costs on them is essential. This paper, therefore, attempts to explore this issue using Chinese private sector enterprise data (CPES) from 2002-to 2019 and to conduct regression analyses on a sample of four major categories: private enterprises, private industrial enterprises, above-scale private industrial enterprises, and below-scale private industrial enterprises. Third, the research methodology is more robust. The paper adopts the double-difference method (DID) to mitigate the endogeneity effect. In addition, the dynamic impact is further explored, and robustness tests are conducted using the counterfactual instrument and event study method so that the results of this paper can more robustly reflect the impact of rising labor costs on firm innovation.

## II. Literature review

### 1. Analysis of the theoretical mechanism of the impact of rising labor costs on firm innovation

On the one hand, rising labor costs can force firms to innovate. [19] argues that labor and capital, as two primary factors of a firm’s production, have a degree of substitution relationship. When there is a relative increase in the price of labor costs, firms will achieve the intended output by increasing the capital factor. Since the minimum wage will force firms to pay higher wages, firms may cut their demand for labor and instead increase their investment in R&D and innovation, replacing labor work with technology and capital. [20] from a demand creation perspective, it argued that rising wages increase consumption through income effects and accelerate the transformation and upgrading of other industries.

The minimum wage plays a role similar to the efficiency wage to some extent [21]. The slack work model suggests that if there is underemployment in the labor market, then an increase in the minimum wage expands the opportunity cost of unemployment, which may force workers to improve their productivity and thus favor firm innovation. [22] suggests that if wages are negotiable and rent-sharing, the rise in wages due to regulatory measures such as the minimum wage system will motivate firms to innovate technologically.

On the other hand, rising labor costs may promote technological innovation through human capital accumulation. Although firms may hire fewer new employees in the face of increasing labor cost forces, they may raise training for existing employees from a firm perspective [22, 23]. In addition, firms can improve their overall human capital by hiring more highly skilled employees to replace low-skilled employees [24].

However, some scholars also argue that rising labor costs may discourage firms from innovating. The increase in labor costs strains the financial position of firms, especially labor-intensive firms, and the rise in labor expenditures may force them to miss out on many NPV-positive investments to fill the capital gap caused by the increase in labor costs, i.e., the rise in labor costs has a "crowding-out effect" on firms’ innovation investments and hinders firms’ innovation [25, 26]. This means that rising labor costs have a "crowding-out effect" on firms’ innovation investments, hindering firms from innovating. Moreover, the long and risky cycle of innovation investment requires continuous financial and human investment, and private firms are more constrained to engage in innovative activities due to the greater risk of survival because of property rights arrangements.

### 2. Results of the empirical study on the impact of rising labor costs on firm innovation

More empirical studies on the relationship between rising labor costs and firms’ technological innovation are needed. First, an increase in the minimum wage causes an increase in the wage level of firms. Second, most studies have concluded that rising labor costs promote firm technological innovation, but heterogeneity exists across industries, regions, and firms [3, 6, 27].

Many scholars have used the database of Chinese industrial firms. [28] used the database of Chinese industrial firms from 1998 to 2007 and found that firms’ innovation capacity rises with increased labor costs. [29] also used the database of Chinese industrial firms from 1998 to 2007, with the local minimum wage as an instrumental variable. The regression results show that wage increases help increase firm productivity, with private and labor-intensive firms being most affected. After the emergence of the shortage of civilian workers in 2004, the impact was greater than before. [30] using a database of Chinese industrial firms from 2005- to 2010 and the FMOLS model, concluded that rising labor costs force exporters to transform and upgrade. Nevertheless, the impact is heterogeneous across industries, regions, and firms. [31] used the database of industrial enterprises for 1998–2007 and firm product-level data from the General Administration of Customs of China for 2000–2006 to study the relationship between labor costs and firm markup rates. The study concluded that firms crossing a certain threshold effect could achieve a dynamic increase in the markup rate through the "process innovation effect" and the "quality upgrade effect"; hence, industry technology level and local institutional factors can affect firms’ innovation behavior.

Many scholars have also used publicly available data on listed companies. [32], A sample of Chinese listed companies from 2002 to 2013 found that technological innovation is an important path to rising labor costs, and it "forces" firms to transform and upgrade. [2], using a sample of manufacturing firms in Shanghai and Shenzhen A-shares for the period 2001–2014, find that rising labor costs promote technological innovation and that stronger employment protection (as measured by minimum wage adjustments) further reinforces this positive effect; however, the effect is weakened by labor intensity as a moderating variable.

However, some scholars have proposed a different perspective. [33] manually collected employee education levels of all A-share listed nonfinancial firms from 2006- to 2013 to investigate whether labor protection promotes innovation among highly educated employees, with innovation indicators derived from the number of patent applications and the number of active patents held in the Patent Research Database of Chinese listed firms from the CSMAR database. The study finds that the mandatory increase in minimum wage significantly reduces the contribution of highly educated employees to firms’ innovation output.

### 3. Policy background: China’s minimum wage regulations

The minimum wage is the lowest remuneration for labor payable by the law. The worker has been offered everyday work during the statutory working hours or the working hours agreed upon in the labor contract stipulated by the law. This system had been in place in China for 27 years when the Ministry of Labour issued the Notice on the Regulations on the Minimum Wage for Enterprises in November 1993. On December 30, 2003, China reissued the Minimum Wage Regulations, taking into account the uneven economic development of different regions and the different living standards of the people, and has therefore tailored the minimum wage levels to local conditions, significantly increasing the minimum wage levels compared to the previous regulations. The minimum wage in China is regulated by the Minimum Wage Regulations, which began on March 1, 2004. The Regulations state that, under normal circumstances, the salaries payable to workers by the employer, excluding wages earned by workers for extended working hours, allowances enjoyed under special conditions such as night work, high temperature, underground work, and poisoning, as well as benefits enjoyed by workers as stipulated by laws, regulations and the State, shall not be lower than the local minimum wage. In case of violation of the rules, the labor security department will order the employer to pay compensation to the workers at one to five times the wages owed. The Regulations also state that the minimum wage standard generally takes the form of a monthly minimum wage standard and an hourly minimum wage standard. The monthly minimum wage rate shall be determined and adjusted based on such factors as the minimum cost of living for employed persons and their dependents in the locality, the consumer price index for urban residents, the social insurance premiums, and housing funds paid by individual employees, the average wage of employees, the level of economic development, and the employment situation. Different administrative regions within the scope of each province, autonomous territory, and municipality directly under the Central Government may have different minimum wage standards.

### 4. Research review

Reviewing the above literature shows that relevant theoretical and empirical studies at home and abroad have been abundant. For example, in China’s labor cost composition, the proportion of wages is around 80%. Therefore, the relationship between the level of salaries paid by enterprises and enterprise innovation can measure the relationship between labor cost and enterprise innovation. Most scholars also estimate the labor cost of enterprises through the wage level of enterprise employees. And the rise in the minimum wage leads to an increase in firms’ labor costs [34]. In conclusion, this paper is a quasi-natural experiment to study the impact of rising labor costs on innovation in private sector firms at the firm level by taking advantage of the "quasi-natural experiment" of the 2004 Minimum Wage Regulation.

In summary, there are several possible mechanisms by which rising labor costs affect firms’ innovation behavior: (1) rising labor costs generate factor substitution effects, which lead firms to choose to use capital and technology as substitutes for labor, thus increasing their investment in innovation; (2) rising labor costs mean that workers’ wage income increases and their consumption may increase accordingly, which expands the market demand for innovation, thus (3) rising labor cost can play the role of elimination of the fittest, and enterprises will innovate to form their core competitiveness in order to survive; (4) rising labor cost not only increases the opportunity cost of employees’ slack work, forcing them to improve productivity, but also makes enterprises hire more capable talents or provide more training to existing employees, which both of which increase the total human capital of enterprises and help improve their innovation capacity; (5) In order to cope with the pressure brought by the increase of minimum wage, enterprises may control the total labor cost by reducing the payment of non-wage benefits, increasing the work intensity of employees, and extending the working hours of employees, or compensate for the increase of labor cost by reducing the investment in R&D, which not only reduces the employees’ incentive to innovate, and will not force enterprises to innovate; (6) the rise in labor costs brought about by the rise in the minimum wage will also reduce enterprises’ external learning opportunities by inhibiting their exports and other forms, which will have a negative impact on their innovation; (7) employees in regions with high minimum wage are better treated, which reduces the risk of unemployment, and according to the lazy person effect, these employees are more likely to settle for the status quo and therefore work effort may be lower; ⑧ according to the equity theory, in regions with high minimum wage, the input-return ratio of hard-working, highly educated employees will further decrease relative to slackers, low-educated or low-wage employees, enhancing the sense of inequity, and therefore work engagement and innovation motivation will also decrease, thus reducing overall innovation output. Among them, ①-④ mechanisms will promote corporate innovation, and ⑤-⑧ mechanisms will hinder it.

In the empirical study, there is still room for further expansion to explore the effect of rising labor costs brought by the minimum wage on firm innovation and its mechanisms.

First, given the different expectations of the above theories and impact mechanisms, we need to test whether rising labor costs promote or hinder firm innovation through empirical studies. Regarding research methodology, only a few domestic scholars have studied the effect of increasing labor costs on enterprise innovation from minimum wage, and the research results differ. Therefore, there is an urgent need for additional relevant research.

Secondly, most domestic studies on the impact of rising labor costs on enterprise innovation use the database of Chinese industrial enterprises and listed companies. Their research objects are mostly state-owned and non-state-owned large and medium-sized industrial enterprises above the scale. Still, less focus is on private enterprises and enterprises below the scale; thus, they cannot measure the impact of rising labor costs on enterprise innovation in a comprehensive way. As there are many small and micro enterprises in China, it is also essential to measure the impact of rising labor costs on them.

## III. Method

### 1. Data source

#### 1.1 China private sector survey data

The Chinese Private Enterprise Survey (CPES) is conducted by the China Federation of Industry and Commerce, the State Administration for Industry and Commerce, and the China National (Private) Economic Research Association and relies on the strength of the provincial (regional, and municipal) industry, commerce federations and industrial and commercial bureaus at a practical level. In addition, experts from the Institute of Sociology of the Chinese Academy of Social Sciences, Beijing Academy of Social Sciences, Beijing University of Technology, and other academic groups and higher education institutions have been involved in the design and writing of the survey since its inception. Furthermore, the Research Centre undertook a specific study for Private Enterprise Owners of the Chinese Academy of Social Sciences.

The survey is a multistage sampling of a certain percentage (approximately 0.05%, varying each time slightly). First, the total number of households to be sampled and the number of homes in each province, municipality, and autonomous region were determined. Second, within each section, municipality, and autonomous region, one planned city or provincial capital city, one prefecture-level city, one county-level city, and one county with high, medium, and low levels of economic development making a total of six cities and counties were determined. Third, the number of urban and rural survey households was selected for the urban and rural areas. Fourth. The number of households surveyed in each industry was determined according to the industry distribution in urban and rural areas. Fifth, specific surveyed households were selected according to the principle of equal spacing. Although the content of each sample survey varies, basic information about business owners and enterprises are fixed survey items to ensure data continuity and comparability.

The China Private Enterprise Survey (CPES) is currently one of China’s longest-running large-scale national sample surveys. Conducted every two years, 14 surveys were born between 1993 and 2019, covering essential information on private enterprises and their owners to ensure data continuity and comparability. In addition, the survey is a multistage sample at a certain nationwide proportion (approximately 0.05%), which is representative and reflects the situation of private enterprises in China more realistically and comprehensively. In this paper, data published from 1995-to 2019 (1995, 1997, 2000, 2002, 2004, 2006, 2008, 2010, 2012, 2014, 2016, and 2018) were selected according to the needs of the study, resulting in a composite panel data containing 12 years of surveys with a total of 29,749 observations. Furthermore, the survey differs from the Chinese Industrial Enterprise Database in that it not only covers industrial enterprises above the scale but also covers enterprises with sales less than 5 million yuan, that is 42.3% of all enterprises, indicating that approximately half of the enterprises are below the scale, which enables us to understand the impact of rising labor costs on private enterprises and enterprises below the scale.

#### 1.2 Census data

In this paper, we use the 2005 census data to calculate the proportion of adolescent workers aged 16–19, 20–24, and 16–24 to the total workforce in each province (municipality, district) (here, we define the entire workforce as the population aged 16 and older in employment) and find the median, decile, and 90th percentile of the data for all three in each province (city, district).

### 2. Variable selection

Table 1 describes the data characteristics of the variables for the entire sample. Among the dependent variables, innovation performance, based on previous literature and data availability, is represented by this paper’s innovation input indicator: R&D expenditure. Since the innovation indicator in this paper is the absolute value of R&D expenditure, which is susceptible to the influence of enterprise size and operating conditions, control variables such as the number of enterprise employees, annual net profit of enterprises, and annual per capita sales amount of enterprises are included in this paper. These data are of considerable magnitude, so logarithmic processing is used. However, the logarithmic treatment does not change the relative relationships between variables in the data.

**Table 1:** Descriptive statistics of full sample variables.

Variable Name	Variable Description	Average value	Standard deviation	Observed values
R&D expenditure	Log of company’s R&D expenditure (million yuan)	1.485	2.854	29749
Number of employees	Company’s annual number of employees (people) log	3.541	1.554	29749
Net profit	Company annual net profit (billion yuan)	0.048	0.271	29749
Sales per capita	Log annual company sales per capita (million yuan)	11.483	1.852	29749
The proportion of the mobile population	Annual mobile population ratio by province	0.182	0.148	29749
Annual average salary	Log yearly average salary by province (yuan)	10.741	0.515	29749
Annual GDP per capita	Log annual GDP per capita by province (yuan)	9.752	0.652	29749

In contrast, it compresses the variables’ scale, making the data smoother and weakening problems such as heteroskedasticity in the model. Since the net profit data of some enterprises are harmful, this paper does not logarithmize the data for it. Nevertheless, to make the magnitude more consistent, this paper sets the unit to billion yuan, which does not change the relative relationships between variables in the data. Controlling for the three variables of the mobile population proportion, average annual wage, and annual GDP per capita at the provincial level reduces the impact of policy bias and economic and social development changes on enterprise R&D expenditures at the local level.

### 3. Empirical strategy

The implementation of the Minimum Wage Regulation is a relatively exogenous policy shock for individual firms, so borrowing the changes in labor costs brought about before and after the implementation of this policy to construct a causal relationship between labor costs and firm innovation can alleviate the endogeneity problem in the empirical study to some extent. Therefore, this paper produces a dual difference equation at the strong level as follows.

$$y_{ict}=\beta_{0}+\beta_{1}\;\mathrm{treat}+\beta_{2}\;\mathrm{period}+\beta_{3}\;\mathrm{treat}\times \mathrm{period}+\alpha_{1}Z_{ict}+\alpha_{2}C_{ct}+\varepsilon_{i}$$ (1)

where the dependent variable *y*_*ict*_ denotes the innovation of firm i in the province (municipality, district) in year t, measured by R&D expenditures, and will be in logarithmic form. *β*_1_ Denotes the gap between the treatment and control groups, *β*_2_ means the impact over time, and *β*_3_ denotes the effects due to the implementation of the Minimum Wage Regulation after controlling for time trends and the effect of the treatment and control groups. We hold firm characteristics and province (city, region) characteristics further.

For the distinction between treatment and control groups, according to the heterogeneous labor force model, adolescents are more affected by the minimum wage than adults because they are usually at the lowest end of the income distribution and a large proportion of low-wage workers are adolescents (Brown, 1993). This paper draws on Card (1992) to measure the ratio of adolescent workers aged 16–19, 20–24, and 16–24 to the total workforce in each province (municipality or district) by using the proportion of firms in that province (municipality or district) that are affected by the Minimum Wage Regulations The size of the impact of the implementation. Theoretically, the more significant the proportion of adolescent workers in a province (municipality or district), the more significant the effect of the Minimum Wage Regulations on the enterprises in that province (municipality or community).

This is done as follows: using the 2005 census data, calculate the proportion of adolescent workers aged 16–19, 20–24, and 16–24 to the total workforce in each province (municipality, district) (where the entire workforce is defined as the population aged 16 or older and in employment status) and find the median, decile and 90th percentile of the corresponding provincial (city and district) data. Then, the enterprises in the provinces (cities and districts) where the ratio is higher than the median are set as the treatment group (treat = l for the treatment group), and the enterprises in the provinces (cities and districts) where the ratio is lower than the median are set as the control group (treat = 0 for the control group). To make the treatment group and the control group more comparable, this paper also tries to set the enterprises in provinces (cities and districts) with a ratio higher than the median and lower than the 90th percentile as the treatment group (treat = l indicates the treatment group), and the enterprises in provinces (cities and districts) with the ratio size lower than the median and higher than the 10th percentile as the control group (treat = 0 indicates the control group), i.e., to carry out the tailing process. The specific grouping the details of the situation are shown in Schedule 1 in S1 Appendix.

Finally, six pairs of treatment and control groups are used in this paper: (1) the treatment group is for enterprises in provinces (cities and districts) where the proportion of adolescent workers aged 16–19 to total workers is the median and above, and the control group is for enterprises in provinces (cities and districts) where the proportion of adolescent workers aged 16–19 to total workers is the median and below; (2) The treatment group was enterprises in provinces (cities and districts) where the proportion of adolescent workers aged 16–19 to total workers was at or above the median and below the 90th percentile, and the control group was enterprises in provinces (cities and districts) where the proportion of adolescent workers aged 16–19 to total workers was at or below the median and above the 10th percentile; (3) the treatment group was enterprises in provinces (cities and districts) where the proportion of 2 enterprises in provinces (cities and districts) where the proportion of adolescent workers aged 0–24 to the total workforce is at the median and above, and the control group is enterprises in provinces (cities and districts) where the proportion of adolescent workers aged 20–24 to the total workforce is at the median and below; (4) the treatment group is enterprises in provinces (cities and districts) where the proportion of adolescent workers aged 20–24 to the total workforce is at the median and below; (5) the treatment group is enterprises where the proportion of adolescent (4) The treatment group is for enterprises in provinces (cities and districts) where the proportion of adolescent workers aged 20–24 to total workers is at or above the median and below the 90th percentile, and the control group is for enterprises in provinces (cities and districts) where the proportion of adolescent workers aged 20–24 to total workers is at or below the median and above the 10th percentile; (5) The treatment group is for enterprises where the proportion of adolescent workers aged 16–24 to total enterprises in provinces (municipalities and districts) where the proportion of adolescent workers aged 16–24 to total workers is at or above the median and the control group is in provinces (municipalities and districts) where the proportion of adolescent workers aged 16–24 to total workers is at or below the median; (6) the treatment group is in provinces (municipalities and districts) where the proportion of adolescent workers aged 16–24 to total workers is at or above the median and below the 90th percentile The control group is for enterprises in provinces (municipalities and districts) where the proportion of adolescent workers aged 16–24 to total workers is at or below the median and above the 10th percentile. Among them, (1), (3), and (5) use the full sample, while (2), (4), and (6) remove the sample of enterprises in the upper and lower 10 percent of provinces (cities and districts). The latter’s advantage is that the control and treatment groups are relatively closer to each other, affected by the minimum wage standard. The effect of extreme values can be excluded, but this may make the sample missing some representativeness. In the specific application and interpretation, this paper will give priority to the full sample data, i.e., the (1), (3), and (5) groups of samples.

## IV. Results and discussion

### 1. The impact of the minimum wage on labor costs

The impact of the minimum wage on the labor costs of enterprises has been a problematic area of research: 93% of enterprises stated that employees would ask for a pay raise because of the increase in the minimum wage. In addition, 68% said that the minimum wage would significantly impact business operations. However, from the above responses from private sector enterprises, it is clear that the increase in the minimum wage has a significant impact on the labor costs of some private sector enterprises, which may affect their investment in innovation resources.

We utilize structural equation modeling (SEM) because of the multiple components of labor costs and the number of influencing factors. In this paper, the change in a firm’s labor costs (latent variable, price) is expressed in terms of four observed variables: wage increases (wage), training costs as a proportion of labor costs (train), changes in social security expenditures (security) and changes in nonwage benefits (welfare). The factors affecting labor cost changes can be divided into internal and external factors. Internal factors include the pay structure of the firm (rmw), the firm’s ability to pay employees (GPR), changes in the size of fixed assets (fix), and changes in the size of the firm’s workforce (worker). We consider these four variables as latent variables (ss) of the internal influences on labor cost. We believe the above four variables are the observed latent variables (ss) of the internal forces on labor costs. External influences include the minimum wage growth rate (MW) and the degree of competition in the industry (income). Our survey of private firms found that government support for innovation in the private sector is primarily ex-post and modest, so it is not considered an external influence here. Since the growth rate of the minimum wage is available and the degree of competition in the industry can be collected using questionnaires, these two variables are used as observable exogenous variables affecting changes in labor costs. To avoid estimation errors arising from the measurement method, we used different data types to design the observed variables for the latent variables.

The SEM output is presented in Table 2, and fully standardized coefficients are reported. Validated factor analysis (CFA) under the total sample found that most loads were more significant than 0. 32 in absolute value, the latent variables were all significant in explaining the observed variables, and the coefficient on MW in the regression was significantly positive. As estimated from the total sample, the faster the minimum wage increases, the more labor costs increase occur in the private sector. To demonstrate the impact of the minimum wage on the labor costs of different types of private enterprises, we divided the enterprises into all private enterprises, above-scale private enterprises, and below-scale private enterprises. The SEM estimation results for each subsample show that the latent variables still mainly explain the observed variables significantly, indicating that the minimum wage increase significantly raises the labor costs of private firms.

**Table 2:** Structural equation output results.

CFA	Observed variables	Full sample	Private enterprises oversize	Private enterprises undersize
lcost	gwage	0. 48***	0.10**	0.15***
	rctrain	0. 02***	0.79***	0.47***
	security	-0.15***	-0.047**	-0.45*
	welfare	0. 74***	0.01	0. 02***
ss	fix	0. 69	0.82	0.48
	gpr	0. 02***	0.45***	0.43***
	nworker	0. 70***	0.41***	0.43***
	rmw	0. 25***	0.10***	0. 74***

Note: Standard deviation of coefficient estimates in parentheses

*** (**, *) indicates significance at the significance level α = 0.01 (0.05,0.1).

### 2. The impact of rising labor costs on private enterprises

In this section, we explore the impact of rising labor costs on innovation in private sector firms across all industries based on the proportion of adolescent workers in different age groups, which is regressed separately in this section, given that this paper uses a double difference model (DID), with the critical explanatory variable being the interaction term *treat period* between the group dummy and the time dummy. The coefficients of these dummy variables indicate the impact of the minimum wage after controlling for time trends and group differences, with the effect due to the implementation of the Minimum Wage Regulation. Standard deviations are clustered to the provincial level, and all regressions control for local fixed products and the industry set effects. Year-fixed effects of reducing the interference of policy bias, changes in economic and social development, and characteristics of the industry at the provincial level. The regression results are shown in Table 2.

In Table 3, the coefficients of the *treatment period* and the remaining control variables in the six regression groups are relatively close in absolute magnitude and generally consistent in sign direction, indicating more robust results. Specifically, the coefficient on *treat period* is positive but not significant when the percentage of adolescent workers aged 16–19 is used to distinguish between the treatment and control groups. On the other hand, the coefficients of *treat period* are significantly positive when the percentage of adolescent workers aged 20–24 and 16–24 are used to distinguish between the treatment and control groups. The proportion of adolescent workers aged 16–19 in the total labor force in all Chinese provinces (cities and districts) is approximately 1. 009% on average, which is a small proportion with relatively minor differences among provinces (cities and districts), while the proportion of adolescent workers aged 20–24 in the total labor force is approximately 2. 348% on average, which is a large proportion with relatively significant differences among provinces (cities and districts). The differences are relatively significant. Therefore, it is indeed more appropriate to use the proportion of the youth labor force aged 20–24 to measure the impact on private enterprises in each province (municipality or district) as a result of the implementation of the Minimum Wage Regulation, which is consistent with the predictions made earlier in this paper. From the estimation results in Columns (3)-(6), it can be seen that the increase in labor cost will encourage the enterprises in the treatment group to increase their innovation investment, and the proportion of their R&D investment will increase by approximately 22.5%-29.4%.

**Table 3:** Impact of rising labor costs on innovation inputs of private firms.

	Treatment group: 16-19-year-olds in the workforce account for more than a fraction		Treatment group: 20-24-year-olds in the workforce account for more than a fraction		Treatment group: 16-24-year-olds in the workforce account for more than a fraction	
	(1)	(2)	(3)	(4)	(5)	(6)
	50–100%	50–90%	50–100%	50–90%	50–100%	50–90%
Treat*period	0.102	0.045	0.240***	0.210**	0.241***	0.615*
	(0.852)	(0.145)	(0.115)	(0.174)	(0.202)	(0.141)
Number of employees	0.552***	0.552**	0.541**	0.5263***	0.526**	0.515***
	(0.040)	(0.063)	(0.029)	(0.015)	(0.023)	(0.358)
Net profit	0.663**	0.524***	0.6415***	0.563**	0.663***	0.526***
	(0.165)	(0.163)	(0.263)	(0.102)	(0.126)	(0.174)
Sales per capita	0.185**	0.856**	0.174**	0.1747**	0.102**	0.126**
	(0.096)	(0.015)	(0.085)	(0.015)	(0.026)	(0.047)
Percentage of mobile population	0.256	0.156	0.041	-0.119	0.082	-0.072
	(0.850)	(0.676)	(0.452)	(0.269)	(0.68)	(0.636)
Annual average salary	0.352	0.249	0.541	0.1802	0.552	0.2748
	(0.336)	(0.385)	(0.602)	(0.345)	(0.348)	(0.412)
Annual GDP per capita	-0.070	-0.371	-0.114	0.052	-0.092	0.0748
	(0.426)	(0.676)	(0.452)	(0.514)	(0.448)	(0.585)
Provincial weekly fixed effect	YES	YES	YES	YES	YES	YES
Firm fixed effect	YES	YES	YES	YES	YES	YES
Year fixed effect	YES	YES	YES	YES	YES	YES
Observed value	21855	21855	21855	21855	21855	21855
R^2^	0.30152	0.3085	0.3085	0.3015	0.3074	0.300

Note: The superscripts

***, **, and * represent significance at the 1%, 5%, and 10% levels, respectively, and standard errors are in parentheses.

Looking at the control variables shows that the larger the size of the private firm (more employees), the more sales per capita, and the more net profit, the greater the firm’s R&D investment (passing the 1% significance level test), which is in line with theoretical expectations and reality. Net profit and firm size (number of employees) have a more significant impact. Pla-Barber also concluded that fit size has a significant contribution to innovation in China. The positive relationship between firm size and innovation originates mainly from nonstate-owned rather than state-owned firms. This argument is consistent with the findings obtained in this section [35].

However, the above is only the average treatment effect of implementing the Minimum Wage Regulation. It does not take into account the dynamic changes of the lagged effect. Therefore, the long-term effects of the performance of the Minimum Wage Regulation, i.e., the gradual impact process of the implementation of the Minimum Wage Regulation, cannot be captured. To examine this dynamic effect, the paper next splits the PERIOD variable in Table 2 into annual dummy variables to explore the marginal impact of the Minimum Wage Provision implementation in each year. Finally, in the regressions, considering the multicollinearity problem, this paper takes 2003, the year closest to implementing the Minimum Wage Regulation, as the reference year. The estimation results are shown in Table 3.

The findings in Table 4 are more consistent with Table 3, where the coefficients of the cross-sectional terms of the group dummy variable and the year dummy variable are insignificant when the percentage of adolescent workers aged 16–19 is used to distinguish between the control and treatment groups, while the coefficients of the cross-sectional terms of the dummy variable are insignificant when the percentage of adolescent workers aged 20–24, as well as 16–24, is used to distinguish between the control and treatment groups. It can be seen that the coefficients of treatx2009 and treatx2011 are significantly positive in Columns (3) and (5) of Table 3. Furthermore, the coefficient of treatx2009 is positive in Columns (4) and (6), indicating that the impact of the implementation of the Minimum Wage Regulation has a significant lagged and cumulative effect, with the fifth year after the implementation year (2009), after the performance of the Minimum Wage Regulation, to significantly promote innovation investment by private firms, from the total sample, and the effect continues until 2011. In particular, the reduced-tailed selection shows private firms’ innovation inputs only in 2009, but no significant cumulative effect is found. We can thus conclude that the positive impact of implementing the Minimum Wage Regulation on private firms’ innovation inputs is relatively lagging. Combined with the descriptive statistics, it can be seen that the R&D investment of private firms experienced a decrease and then an increase after the implementation of the Minimum Wage Regulation, indicating that the performance of the Minimum Wage Regulation will go through a more prolonged and more painful process before it has a positive effect on firms’ innovation.

**Table 4:** Dynamic impact results.

	Treatment group: 16-19-year-olds in the workforce account for more than a fraction		Treatment group: 20-24-year-olds in the workforce account for more than a fraction		Treatment group: 16-24-year-olds in the workforce account for more than a fraction	
	(1)	(2)	(3)	(4)	(5)	(6)
	50–100%	50–90%	50–100%	50–90%	50–100%	50–90%
Treat*2001	-0.148	0.051	-0.248	-0.102	-0.202	-0.102
	(0.215)	(0.140)	(0.135)	(0.147)	(0.1445)	(0.153)
Treat*2005	0.015	0.0002	0.1154	0.174	0.1502	0.002
	(0.110)	(0.145)	(0.1115)	(0.025)	(0.152)	(0.174)
Treat*2007	-0.052	0.074	0.1150	0.102	0.102	0.348
	(0.174)	(0.179)	(0.115)	(0.152)	(0.102)	(0.525)
Treat*2009	0.002	0.002	0.312**	0.521**	0.282**	0.283*
	(0.174)	(0.115)	(0.141)	(0.011)	(0.102)	(0.152)
Treat*2011	0.185	0.110	0.2562*	0.202	0.248*	0.2265
	(0.170)	(0.174)	(0.185)	(0.263)	(0.126)	(0.145)
Number of employees	0.556**	0.541**	0.565**	0.512**	0.541**	0.155**
	(0.045)	(0.518)	(0.026)	(0.590)	(0.026)	(0.9859)
Net profit	0.662**	0.526	0.602**	0.559**	0.603**	0.559**
	(0.174)	(0.126)	(0.958)	(0.1465)	(0.148)	(0.174)
Sales per capita	0.185**	0.1851***	0.183**	0.141	0.152**	0.125**
	(0.002)	(0.015)	(0.013)	(0.966)	(0.013)	(0.074)
Percentage of mobile population	0.202	0.215	0.1263	0.1625	0.2752	0.226
	(0.452)	(0.752)	(0.3285)	(0.541)	(0.469)	(0.706)
Annual average salary	0.3052	0.390	0.76	0.323	0.705	0.372
	(0.359)	(0.463)	(0.36)	(0.385)	(0.343)	(0.387)
Annual GDP per capita	-0.071	-0.385	0263	0.166	0.1852	0.0152
	(0.410)	(0.6726)	(0.4634)	(0.426)	(0.452)	(0.567)
Provincial weekly fixed effect	YES	YES	YES	YES	YES	YES
Firm fixed effect	YES	YES	YES	YES	YES	YES
Year fixed effect	YES	YES	YES	YES	YES	YES
Observed value	21885	15402	21885	15205	21885	14996
R^2^	0.3056	0.3062	0.3052	0.3026	0.3026	0.3052

Note: The superscripts ***, **, and * represent significance at the 1%, 5%, and 10% levels, respectively, and standard errors are in parentheses.

On June 29, 2007, the Standing Committee of the Tenth National People’s Congress adopted the Labour Contract Law at its twenty-eighth meeting, which came into force on January 1, 2008. Article 20 of this law states that "the wages of workers during the probationary period shall not be less than the minimum wage for the same position in the unit or 80 percent of the wage agreed in the labor contract and shall not be less than the minimum wage standard in the employer’s locality". Thus, the Labour Contract Law can strengthen the enforcement of the minimum wage system to a certain extent. However, the pressure of rising labour costs is also more significant for enterprises, and the demand for enterprise innovation is further increased. Thus, in 2009, the positive impact of increasing labor costs on firms’ innovation investment was revealed by combining both.

**Fig 1:**
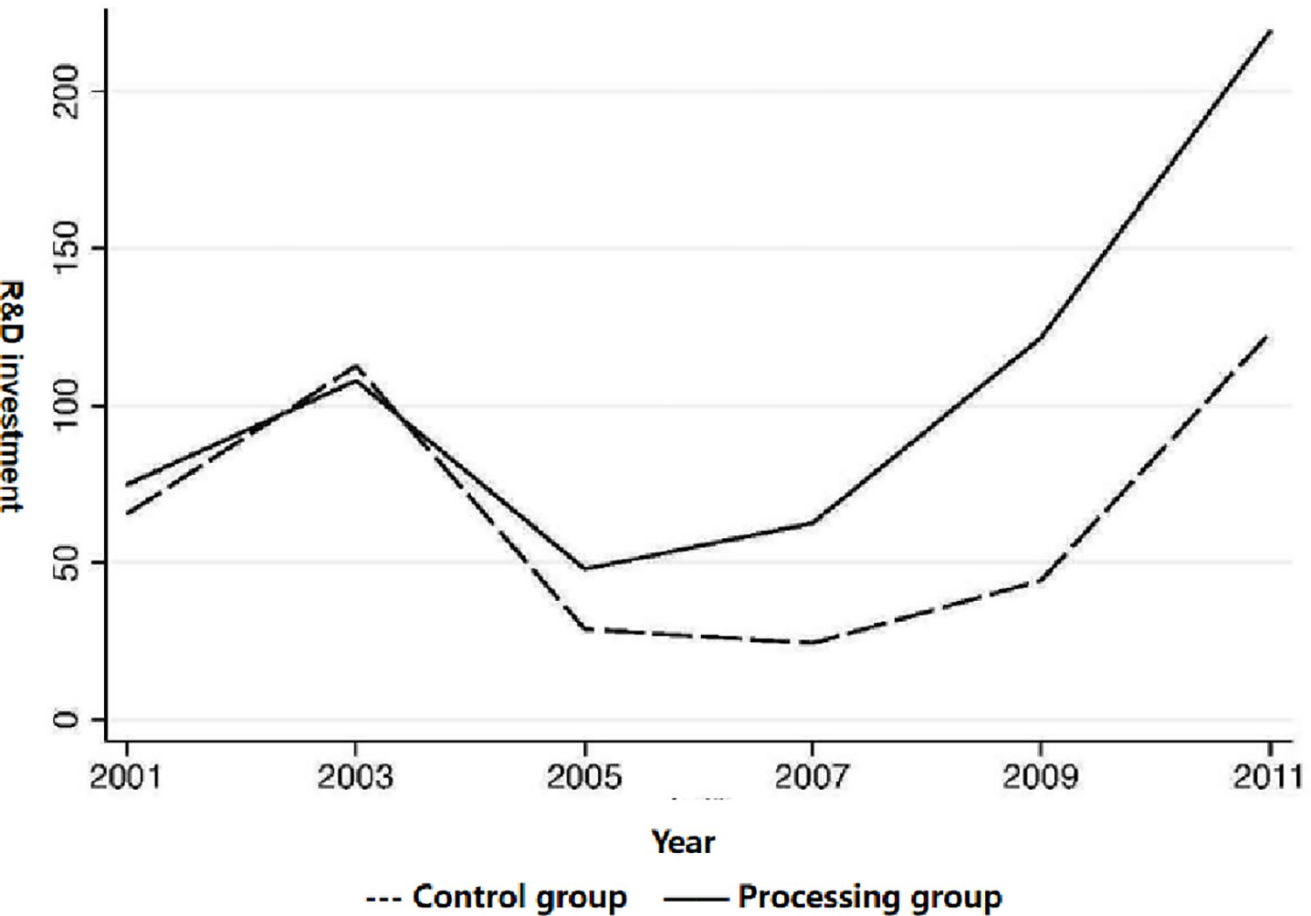
Trends in the evolution of R&D investment of enterprises in the treatment and control groups (unit: Million yuan).

On the other hand, a strong identification constraint of the double-difference method (DID) is that before the official implementation of the Minimum Wage Regulation (i.e., 2004), firms in the treatment and control groups were supposed to satisfy the parallel trend assumption. Fig 1 portrays this trend using the share of adolescent workers aged 20–24 as an example of distinguishing between the treatment and control groups. After implementing the Minimum Wage Regulation in 2004, the R&D investment of the treatment and control groups declined 2005. Nevertheless, the treatment group was affected by the Minimum Wage Regulation to a lesser extent than the control group. In 2007, the R&D investment of the treatment group started to rebound, while the R&D investment of the control group slightly decreased. In 2009 and 2011, R&D investment increased significantly in both the treatment and control groups, but the treatment group started to rebound earlier and rose more.

Meanwhile, the regression results in Columns (1)-(6) of Table 4 further verify the parallel trend. Furthermore, the coefficient of treatx2001 is not significant, indicating that before the implementation of the Minimum Wage Regulation (2004), the treatment and control group firms were comparable. Thus, there was no sample selection problem, i.e., the significance of the impact of the Minimum Wage Regulation implementation was not affected by the endogenous grouping, the parallel trend between the treatment and control groups was primarily satisfied, and the primary estimation results were reliable. Therefore, implementing the Minimum Wage Regulation will promote corporate innovation in firms that are more affected by it, and the findings of this paper are robust.

### 3. The impact of rising labor costs on private industrial enterprises

This section explores the impact of rising labor costs on innovation in private industrial firms. Most previous studies on increasing labor costs on firm innovation have used the Chinese industrial enterprise database. Industry includes three industries: mining, manufacturing, electricity, gas, and water production and supply. Theoretically, when labor is relatively abundant, and wages are low, industrial enterprises have less demand for technological innovation. However, when labor costs rise, industrial enterprises’ need for innovation increases accordingly, and they are more motivated to engage in technological innovation. In addition, previous scholars have mainly studied industrial enterprises above the scale but rarely studied industrial enterprises below the scale. At the same time, this paper attempts to understand the influence of corporate innovation on rising labor costs for industrial enterprises of different scales. Specifically, this paper defines industrial enterprises with an annual primary business income of 5 million RMB and above as above-scale industrial enterprises.

In comparison, industrial enterprises with a primary yearly income of less than 5 million RMB are defined as below-scale industrial enterprises according to the National Bureau of Statistics criteria before 2011. A subsample study is conducted again. Table 4 reports the results of the tests of the effects and dynamic effects of the implementation of the Minimum Wage Regulation on the innovation inputs of all private industrial enterprises, above-scale private industrial enterprises, and below-scale private industrial enterprises.

Table 5 shows that the coefficient of the treatment period is significantly positive at the 5% confidence level for all private industrial firms. The absolute value of the coefficient (0.348) is greater than the total value of the coefficient *treat period* in Table 5 (0.294). The coefficient of treatX2009 is also significantly positive at the 5% confidence level, and the absolute value is greater than the coefficient of treatX2009 in Table 3. The coefficient of treatX2009 is also significantly positive at a 5% confidence level, and the absolute value is also more significant than the coefficient of treatX2009 in Table 3. At the same time, the cross-terms of treatment with other years are not necessary. This indicates that the rise in labor costs has a more positive effect on the innovation input of private industrial firms; these firms are more responsive to the increase in labor costs and more motivated to engage in technological innovation than firms in other industries. Moreover, private industrial firms suffer the same lagged effect as those in the remaining industries.

**Table 5:** Test of the impact on innovation inputs of private industrial enterprises and their dynamic effects.

	All private industrial enterprises		Above-scale private industrial enterprises		Private industrial enterprises under the scale	
	(1)	(2)	(3)	(4)	(5)	(6)
Treat*period	0.34**		0.459**		-0.241	
	(0.418)		(0.1159)		(0.149)	
Treat*2001		-0.3596		-0.525		-0.156
		(0.203)		(0.302)		(0.052)
Treat*2005		0.139		0.056		0.242
		(0.2093)		(0.203)		(-0.026)
Treat*2007		0.1559		0.023		-0.026
		(0.126)		(0.226)		(0.226)
Treat*2009		0.459**		0.5952*		0.148
		(0.185)		(0.259)		(0.245)
Treat*2011		0.105		0.159		0.085
		(0.226)		(0.262)		(0.255)
Provincial weekly fixing effect	YES	YES	YES	YES	YES	YES
Firm fixed effect	YES	YES	YES	YES	YES	YES
Year fixed effect	YES	YES	YES	YES	YES	YES
Observed value	8495	8256	5685	5685	2954	2949
R^2^	0.326	0.352	0.3526	0.352	0.362	0.3052

Note: The superscripts

***, **, and * represent significance at the 1%, 5%, and 10% levels, respectively, and standard errors are in parentheses.

The results for the above-scale private industrial enterprises are similar to those estimated for all enterprises. Nevertheless, the estimated coefficients of the treatment period and treatX2009 are more significant in absolute value at 0.406, indicating that private industrial enterprises above the size are more positively affected by the rise in labor costs. This result is consistent with the findings of the currently available empirical studies. However, the estimated coefficients of the treatment period and treatX2009 are not significant for subscale private industrial enterprises, indicating that subscale private industrial enterprises are not significantly affected by the rising labor cost. This paper suggests that a possible reason is the low absolute value and slight differences in innovation inputs of subscale private industrial firms. In the sample of this paper, the average R&D investment of private industrial enterprises above the scale is 2.1915 million yuan, and the average annual wage cost is approximately 5.35 million yuan.

In comparison, the average R&D investment of private industrial enterprises below the scale is only 20.59 million yuan, and the average annual wage cost is approximately 535,000 yuan. Thus, whether it is R&D investment or average annual wage cost, the former is more than ten times the latter. In addition, the smaller the size of the enterprise is, the worse the financing capacity. Therefore, the more limited and riskier the innovative activities are, the less likely it is for a firm to engage in technological and product innovation. Thus, labour costs are not an essential factor influencing the R&D investment decisions of subscale private industrial firms.

### 4. Robustness test

#### 4.1 Placebo test

Next, the paper conducts a placebo test by randomly varying the sample selection of the treatment and control groups and the year of implementation of the Minimum Wage Regulation.

**Fig 2:**
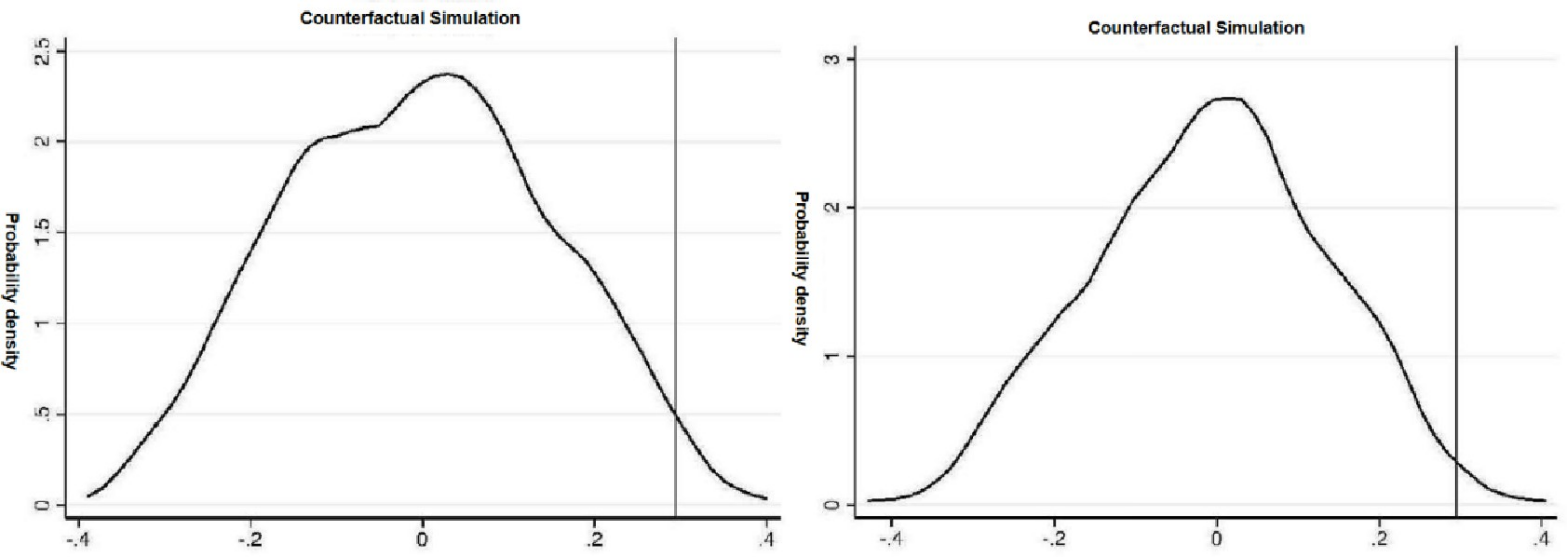
Placebo test for randomly selected groups (500 times).

First, the treatment and control groups’ sample selection was randomly changed for the placebo test. Specifically, firms in 15 provinces (cities and districts) from the total sample of 30 areas (cities and communities) (excluding Tibet Autonomous Region and Taiwan Province) were randomly selected as the treatment group. Firm SMS in the other 15 provinces (cities and districts) were used as the control group to construct the counterfactual instrumental variable *treatfalsexperiod*, which theoretically would not significantly affect firms’ innovation behavior and thus would not affect firms’ R&D investment. Therefore, there should not be a significant difference between the R&D investment of enterprises in the treatment and control groups in the placebo test. To avoid the interference of chance events with the conclusion, this paper conducted 500 random simulations to derive the distribution of the *treatfalseXperiod* coefficient (see Fig 2).

In Fig 2, the vertical line indicates the actual value of the coefficient of the *treatment period*, and the curve shows the distribution of the coefficient of the *treat false—a period* in the random simulation. The actual value of the coefficient of treat*period (vertical line) is located on the right side of the distribution of counterfactual coefficients instead of in the middle (near the value of 0). Furthermore, 500 random simulation regressions of treat also period coefficients were smaller than the actual values for 497 times. Thus, the placebo test with randomly selected groupings suggests that rising labor costs positively promote firms’ innovation inputs.

#### 4.2 Event study method test

The event study method was first invented and used in modern financial econometrics. However, the most widely used and fruitful area is the study of corporate finance, which is mainly used to measure the impact of an event on a company’s stock price. The basic idea and steps are as follows: (1) assume a counterfactual "normal return," which is usually estimated using mean-adjusted, market-adjusted, and market-model methods; (2) calculate the actual return; (3) calculate the difference between the actual return and the average return, i.e., the excess return; and (4) if the event does not affect the company’s stock price, the excess return should be white noise, which can be tested by constructing a t-statistic.

First, a "stationarity test" is performed using the 2001 and 2003 samples. Specifically, this paper generates cross-terms of all explanatory variables and groups dummy variables (treat) into the equation for a two-period mixed OLS regression. The coefficients of all cross-sectional variables were insignificant, indicating no significant difference in R&D investment between the treatment and control groups before implementing the Minimum Wage Regulation and the "stationarity test" was passed.

In the second step, this paper performs mixed OLS estimation using the control group samples of 2005, 2007, 2009, and 2011 to obtain the estimated coefficients of each explanatory variable and multiply these coefficients by the explanatory variables of the treatment groups of 2005, 2007, 2009 and 2011 accordingly to obtain the estimated $\hat{\ln rd}(\mathrm{yhat})$.

Finally, a t-test is used to confirm whether there is a significant difference between $\hat{\ln rd}(\mathrm{yhat})$ and the actual lnrd of the treatment group. If there is a considerable difference, the Minimum Wage Regulation does affect the R&D investment of the enterprises in the treatment group. After the t-test Pr(*T*>*t*) = 0, the original hypothesis is firmly rejected. This indicates that the rising labor cost impacts the R&D investment of enterprises in the treatment group, and the regression in this paper is robust. Since the regression results conducted by this method do not focus on the magnitude of the coefficients and their directions, the specific regression results are not presented in this paper. Similarly, for all private industrial enterprises, above-scale private industrial enterprises, and below-scale private industrial enterprises, the same event study method is tested in this paper, and the results pass the test.

## V. Conclusions and recommendations

### 1. Conclusion

This paper uses the China Private Enterprise Survey (CPES) and other relevant databases to provide descriptive statistics on the temporal evolution of R&D investment in private sector firms of different industries and sizes and indirectly investigates the impact of rising labor costs on innovation investment in private sector firms through a double difference model (DID) using a "quasi-natural experiment" with the official implementation of the Minimum Wage Regulation in 2004. The impact of rising labor costs on private-sector firms’ innovation investment is indirectly investigated through a double difference model (DID).

The estimation results show that for all private firms, the R&D investment of firms in the treatment group increases significantly by approximately 22.5% to 29.4% after implementing the Minimum Wage Regulation. After testing for dynamic effects, this paper finds that this effect has a significant lagged and cumulative impact: the boost was revealed in 2009 (i.e., the fifth year) after the implementation of the Minimum Wage Regulation on March 1, 2004; the effect continues until 2011 for the entire sample. Based on this, we can conclude that the positive impact of implementing the Minimum Wage Regulation on innovation inputs in the private sector is relatively lagging. Combined with the descriptive statistics, we can see that after the implementation of the Minimum Wage Regulation, the R&D investment of private enterprises experienced a process of decrease first and then increase, indicating that the performance of the Minimum Wage Regulation will experience a more prolonged and more painful process before it has a positive effect on the innovation investment of private enterprises. The official implementation of the Labour Contract Law on January 1, 2008, further clarified the wage standard for workers, which strengthened the enforcement of the minimum wage system to a certain extent. As a result, the pressure of rising labour costs is even more significant for enterprises, and enterprises’ demand for innovation increases. Thus, in 2009, the positive impact of increasing labor costs on firms’ innovation inputs was revealed by the combined effect of both. For all private industrial enterprises, the results are similar.

Nevertheless, the absolute value of the coefficient of the critical explanatory variable *treat period* is more significant. The cumulative increase in R&D investment of enterprises in the treatment group is 34.8%, subject to the same lagged effect as for the rest of the private enterprises in the industry. For private industrial enterprises above the scale, the cumulative increase in R&D investment of the treated group enterprises is 40.6%. This indicates that the rising labor cost has played a more positive role in the innovation investment of private industrial enterprises (especially above-scale private industrial enterprises) and that private industrial enterprises (especially above-scale private industrial enterprises) are more responsive to the rising labor cost and more motivated to carry out technological innovation than other private enterprises. In contrast, the R&D investment of private industrial enterprises below the scale is not significantly affected by the rising labor cost.

### 2. Suggestions

1. Develop multi-level education and improve professional skills training. In general, Chinese citizens complete nine years of compulsory education at the age of 16 (or 17), high school education at the age of 19 (or 20), and higher education to get a bachelor’s degree at the age of 23 (or 24). However, the empirical findings show that many adolescent workers are still in China. Most teenage workers aged 16–24 have not completed higher education, and some have even completed only nine years of compulsory schooling. Therefore, it is necessary to do an excellent job of multi-level teaching: based on ensuring universal mandatory nine-year education, workers who cannot receive higher education should be guided to participate in vocational and technical education, vocational skills training, and re-employment training to improve their human capital level and lay a good foundation of talents for enterprise innovation.
2. Improve the way of policy implementation, and classify and precisely apply policies. In adjusting the minimum wage standard, each place will generally be introduced by the local human resources and social security department with the consent of the people’s government of that province and reported to the Ministry of Human Resources and Social Security for the record. The local human resources and social security department will announce 3–5 minimum wage standard grades total governing cities, counties, and states to choose from, and each city, county, and State may select the monthly minimum wage standard and hourly minimum wage standard applicable to its region according to the actual local situation minimum wage standard and hourly minimum wage standard, and report to the provincial office for record. Therefore, when formulating policies, regional human resources and social security departments should conduct in-depth research, go into enterprises to understand the opinions of workers and enterprises, and formulate minimum wage standards suitable for the specific conditions of each city, county, and State, to protect the rights and interests of workers, but also to make the sustainable development of enterprises and enhance the competitiveness of enterprises.
3. Reducing taxes and burdens to lower the cost burden of private enterprises to carry out technological innovation. In the long run, the implementation of the Minimum Wage Regulation does promote the innovation investment efforts of private enterprises that are more affected by it. Still, the process is painful and more prolonged, and labor protection policies such as the Minimum Wage Regulation may deal a more severe blow in the short term. Thus, governments should introduce and implement corresponding supporting measures for outstanding innovative enterprises, providing preferential policies such as land, office space, and taxation. In addition, for exceptional talents within the enterprise, give preferential policies such as subsidies for talent settlement and placement of family members; accelerate the establishment of talent attraction, talent incentive, and talent protection mechanisms, improved infrastructure of all kinds, and social security systems to reduce the cost of talent attraction for enterprises and help them retain high-quality talent.

## Supporting information

S1 Dataset Minimal anonymized data set. (XLSX)

S1 Appendix (DOCX)

## References

[1] Hallegatte Stéphane. ECONOMICS The rising costs of hurricanes. Nat Clim Chang. 2012;2: 148–149.

[2] Perri A, Andersson U, Nell PC, Santangelo GD. Balancing the trade-off between learning prospects and spillover risks: MNC subsidiaries’ vertical linkage patterns in developed countries. J World Bus. 2013;48: 503–514.

[3] Yi HA, Ls B, Gw C. How did rising labor costs erode China’s global advantage? J Econ Behav Organ. 2021;183: 632–653.

[4] Ghiglino C, Venditti A. Wealth inequality, preference heterogeneity and macroeconomic volatility in two-sector economies. J Econ Theory. 2007;135: 414–441.

[5] Santos J, Domingos T, Sousa T, St Aubyn M. Useful Exergy Is Key in Obtaining Plausible Aggregate Production Functions and Recognizing the Role of Energy in Economic Growth: Portugal 1960–2009. Ecol Econ. 2018;148: 103–120.

[6] Nauenberg Eric. Changing healthcare capital-to-labor ratios: evidence and implications for bending the cost curve in Canada and beyond. Int J Heal Care Financ Econ. 2014;14: 339–353. doi: 10.1007/s10754-014-9154-9 25129110

[7] Sarriera JM, Salvucci FP, Zhao J. Worse than Baumol’s disease: The implications of labor productivity, contracting out, and unionization on transit operation costs. Transp Policy. 2018;61: 10–16.

[8] Amable B, Chatelain JB. Can financial infrastructures foster economic development? J Dev Econ. 2001;64. doi: 10.1016/S0304-3878(00)00147-4

[9] Pfeifer C. Adjustments of wage-tenure profiles with respect to entry age. J Bus Econ. 2013;83: 171–191.

[10] Tian X, Yi F, Yu X. Rising cost of labor and transformations in grain production in China. China Agric Econ Rev. 2019;ahead-of-p.

[11] Yang DT, Chen VW, Monarch R. Rising Wages: Has China Lost Its Global Labor Advantage? Pacific Econ Rev. 2010;15.

[12] You K, Sarantis N. Structural breaks and the equilibrium real effective exchange rate of China: A NATREX approach. China Econ Rev. 2012;23.

[13] Mitze T, Schmidt TD. Internal migration, regional labor markets and the role of agglomeration economies. Ann Reg Sci. 2015;55: 61–101.

[14] A MA B AK C AT,. On the connectivity properties and energy of Fibonomial graphs—ScienceDirect. Discret Appl Math. 2014;169: 1–8.

[15] Moritz M, Handa S, Chen YJ, Xiao N. Herding Contracts and Pastoral Mobility in the Far North Region of Cameroon. Hum Ecol. 2015;43: 1–11.

[16] Jefferson G, Albert HU, Guan X, Xiaoyun YU. Ownership, performance, and innovation in China’s large- and medium-size industrial enterprise sector. China Econ Rev. 2003;14: 89–113.

[17] Price L, Wang X, Jiang Y. The challenge of reducing energy consumption of the Top-1000 largest industrial enterprises in China. Energy Policy. 2010;38: 6485–6498.

[18] Sethi D, Judge WQ, Sun Q. FDI distribution within China: An integrative conceptual framework for analyzing intra-country FDI variations. Asia Pacific J Manag. 2011;28: 325–352.

[19] Hicks JR. The Theory of Wages. The Theory of Wages; 1963.

[20] Trindade V. The big push, industrialization and international trade: The role of exports. J Dev Econ. 2005;78: 22–48.

[21] Yashiv E. The determinants ofequilibrium unemployment. Am Econ Rev. 2000;90: 1297–1322.

[22] Allen RC. Engels’ pause: Technical change, capital accumulation, and inequality in the british industrial revolution. Explor Econ Hist. 2009;46: 418–435.

[23] Zwick T. Seniority Wages and Establishment Characteristics. Labour Econ. 2011;18: 853–861.

[24] Campolieti M, Gunderson M, Lee B. The (Non) Impact of Minimum Wages on Poverty: Regression and Simulation Evidence for Canada. J Labor Res. 2012;33: 287–302.

[25] Henningsen MS, Haegeland T, Moen J. Estimating the additionality of R&D subsidies using proposal evaluation data to control for research intentions. J Technol Transf. 2015;40: 227–251.

[26] Hottenrott H, Lopes-Bento C. (International) R&D collaboration and SMEs: The effectiveness of targeted public R&D support schemes. Res Policy. 2014;43: 1055–1066.

[27] Golish BL, Besterfield-Sacre ME, Shuman LJ. Comparing Academic and Corporate Technology Development Processes. J Prod Innov Manag. 2008;25: p.47–62.

[28] Gan L, Hernandez MA, Ma S. The higher costs of doing business in China: Minimum wages and firms’ export behavior. J Int Econ. 2016;100: 81–94.

[29] Bartolini S, Bonatti L. Endogenous growth, decline in social capital and expansion of market activities. J Econ Behav Organ. 2008;67: 917–926.

[30] Li K, Lin B. Impact of energy technology patents in China: Evidence from a panel cointegration and error correction model. Energy Policy. 2016;89: 214–223.

[31] Li SF, Zhu HM, Yu K. Oil prices and stock market in China: A sector analysis using panel cointegration with multiple breaks. Energy Econ. 2012;34: 1951–1958.

[32] Peretto PF, Valente S. Resource Wealth, Innovation and Growth in the Global Economy. J Monet Econ. 2010;58: 387–399.

[33] Zhang M, Wang Y, Zhao Q. Does participating in the standards-setting process promote innovation? Evidence from China. China Econ Rev. 2020;63: 101532.

[34] Brown C. Minimum wages, employment, and the distribution of income. Handb Labor Econ. 1999;3, part b: 2101–2163.

[35] Pla-Barber J, Alegre J. Analysing the link between export intensity, innovation and firm size in a science-based industry. Int Bus Rev. 2007;16: 275–293.

