# Supplementary material for: Can rising labor costs boost private sector R&D investment? : Evidence from a survey of Chinese private firms
Source: PLoS One. 2022 Aug 4;17(8):e0268287. doi: 10.1371/journal.pone.0268287 (PMC9351998; doi:10.1371/journal.pone.0268287)
Supplement: S1 Appendix — (DOCX) [file pone.0268287.s002.docx]

**Appendix**

**Schedule 1. Detailed table of treatment and control groupings**

| Grouping criteria | Grouping number | (treat=1) | (treat=0) |
| --- | --- | --- | --- |
| Percentage of workers aged 16-19 | (1) Full sample grouping | Tianjin, Hebei, Jiangsu and Zhejiang Provinces, Fujian, Shandong, Henan, Hunan, Guangdong, Guizhou and Yunnan Provinces | Beijing, Shanxi, Inner Mongolia Autonomous Region, Jilin, Heilongjiang, Shanghai, Anhui, Jiangxi, Hubei, Guangxi Zhuang Autonomous Region, Hainan, Chongqing, Sichuan, Shaanxi, Gansu, Qinghai, Ningxia Hui Autonomous Region, Uyghur Autonomous Region |
|  | (2) Ending treatment grouping | Tianjin, Hebei Province, Jiangsu Province, Zhejiang Province, Shandong Province, Hunan Province, Liuzhou Province, Yunnan Province | Beijing, Shanxi, Liaoning, Shanghai, Anhui, Jiangxi, Hubei, Guangxi Zhuang Autonomous Region, Hainan, Sichuan, Shaanxi, Gansu, Qinghai, Ningxia Hui |
| Percentage of workers aged 20-24 | (3) Full sample grouping | Beijing, Tianjin, Hebei, Shanghai, Jiangsu, Zhejiang, Fujian, Shandong, Hunan, Guangdong, Hainan, Yunnan and Ningxia Hui Autonomous Region | Shanxi Province, Mongolia Autonomous Region, Liaoning Province, Yanlin Province, Heilongjiang Province, Anhui Province, Jiangxi Province, Henan Province, Hubei Province, Guangxi Zhuang Autonomous Region, Zhuangqing City, Sichuan Province, Guizhou Province, Shaanxi Province, Gansu Province |
|  | (4) Ending treatment grouping | Beijing, Tianjin, Hebei, Jiangsu, Zhejiang, Shandong, Hunan, Hainan, Yunnan, Ningxia Hui Autonomous Region | Shanxi Province, Inner Mongolia Autonomous Region, Jiangxi Province, Henan Province, Hubei Province, Guangxi Zhuang Autonomous Region, Sichuan Province, Guizhou Province, Shaanxi Province, Gansu Province, Xinjiang Uygur Autonomous Region |
| Percentage of workers aged 16-24 | (5) Full sample grouping | Beijing, Tianjin, Hebei, Shanghai, Jiangsu, Zhejiang, Fujian, Shandong, Hunan, Guangdong, Hainan, Yunnan | Shanxi Province, Inner Mongolia Autonomous Region, Liaoning Province, Jilin Province, Heilongjiang Province, Ancient Province, Jiangxi Province, Henan Province, Hubei Province, Guangxi Zhuang Autonomous Region, Zhazhou Province, Shaanxi Province, Gansu Province, Qinghai Province, Ningxia Hui Autonomous Region |
|  | (6) Ending treatment grouping | Beijing, Tianjin, Hebei, Shanghai, Jiangsu, Zhejiang, Fujian, Shandong, Hunan, Hainan, Yunneng | Shanxi Province, Anhui Province, Henan Province, Hubei Province, Guangxi Zhuang Autonomous Region, the four provinces, Huangzhou Province, Shaanxi Province, Gansu Province, Ancient Sea Province, Ningxia Hui Autonomous Region |
